# Nutrition education and leadership for improved clinical outcomes: training and supporting junior doctors to run ‘Nutrition Awareness Weeks’ in three NHS hospitals across England

**DOI:** 10.1186/1472-6920-14-109

**Published:** 2014-05-29

**Authors:** Sumantra Ray, Celia Laur, Pauline Douglas, Minha Rajput-Ray, Mike van der Es, Jean Redmond, Timothy Eden, Marietta Sayegh, Laura Minns, Kate Griffin, Colin McMillan, Alfred Adiamah, Stephen Gillam, Joan Gandy

**Affiliations:** 1 The Need for Nutrition Education/Innovation Programme (NNEdPro) Group, Cambridge University Hospitals & Medical Research Council (MRC) Human Nutrition Research (HNR), Elsie Widdowson Laboratory, 120 Fulbourn Road, Cambridge CB1 9NL, England

**Keywords:** Nutrition, Education, Medical, Doctors

## Abstract

**Background:**

One in four adults are estimated to be at medium to high risk of malnutrition when screened using the ‘Malnutrition Universal Screening Tool’ upon admission to hospital in the United Kingdom. The *Need for Nutrition Education/Education Programme (NNEdPro)* Group was developed to address this issue and the *Nutrition Education and Leadership for Improved Clinical Outcomes (NELICO)* is a project within this group.

The objective of NELICO was to assess whether an intensive training intervention combining clinical and public health nutrition, organisational management and leadership strategies, could equip junior doctors to contribute to improvement in nutrition awareness among healthcare professionals in the National Health Service in England.

**Methods:**

Three junior doctors were self-selected from the NNEdPro Group original training. Each junior doctor recruited three additional team members to attend an intensive training weekend incorporating nutrition, change management and leadership. This equipped them to run nutrition awareness weeks in their respective hospitals. Knowledge, attitudes and practices were evaluated at baseline as well as one and four months post-training as a quality assurance measure. The number and type of educational events held, pre-awareness week Online Hospital Survey results, attendance and qualitative feedback from training sessions, effectiveness of dissemination methods such as awareness stalls, Hospital Nutrition Attitude Survey results and overall feedback were also used to determine impact.

**Results:**

When the weighted average score for knowledge, attitudes and practices at baseline was compared with four months post-intervention scores, there was a significant increase in the overall score (p = 0.03). All three hospital teams conducted an effective nutrition awareness week, as determined by qualitative data collected from interviews and feedback from educational sessions.

**Conclusion:**

The NELICO project and its resulting nutrition awareness weeks were considered innovative in terms of concept and content. It was considered useful, both for the junior doctors who showed improvement in their nutrition knowledge and reported enthusiasm and for the hospital setting, increasing awareness of clinical and public health nutrition among healthcare professionals. The NELICO project is one innovative method to promote nutrition awareness in tomorrow’s doctors and shows they have the enthusiasm and drive to be nutrition champions.

## Background

In 2011, the British Association for Parenteral and Enteral Nutrition (BAPEN) estimated that one in four adults were at medium to high risk of malnutrition when screened using the ‘Malnutrition Universal Screening Tool’ (‘MUST’) upon admission to a hospital in the United Kingdom (UK) [1-3]. Increased awareness on the detection, prevention and management of hospital malnutrition could help reduce its burden due to associated problems including delayed recovery, increased length of hospital stay, worsening of prognosis and an increased risk of serious complications of illness [4]. In 2013, Leach *et al.* showed that although guidelines have been issued by the National Institute for Health and Clinical Excellence (NICE) regarding nutritional screening, dietary requirements and the care of people identified as at risk, these guidelines are often not met. This study also indicated some of the factors which may contribute to this issue including: *‘poor training, lack of time, competing tasks, and a perception that basic care is less important than other duties or the responsibility of others.’*[5]. The *Need for Nutrition Education/Innovation Programme* (NNEdPro) aims to address some of these issues, alongside other key themes in clinical and public health nutrition.

The NNEdPro Group was developed in 2007 as an independent educational innovation and evaluation programme. It arose from the work of the Council of Europe Alliance (UK) on Hospital Food and Nutritional Care, and has at its core the improvement of nutrition education for ‘Tomorrow’s Doctors’ [6]. The *Nutrition Education and Leadership for Improved Clinical Outcomes* (NELICO) project stemmed from earlier work completed by the NNEdPro Group. The original work of the group looked into the effectiveness and value of providing a short training in clinical nutrition to medical students [6-9]. NELICO aimed to assess whether an intensive training intervention could equip junior doctors to run a hospital Nutrition Awareness Week (NAW) and thus contribute to improvement in clinical nutrition and patient outcomes in UK hospitals.

The principal aims of NELICO included:

A. To assess whether providing foundation doctors with a two-day course covering previous (NNEdPro) teaching in clinical nutrition as well as additional training in leadership and change management techniques can result in:

(i) An improvement in knowledge, attitudes and practices (KAP) related to this training intervention.

(ii) The ability and motivation to run a Nutrition Awareness Week (NAW) in their hospital.

B. To evaluate, through staff interviews and feedback, whether a range of National Health Service (NHS) staff believe that the project described above has the potential to contribute to cost savings for the hospital, due to better nutritional care.

C. To gain insight into barriers to, and solutions for, improving awareness and practices relating to nutritional care in the hospital setting.

## Methods

### Recruitment

**Figure 1:**
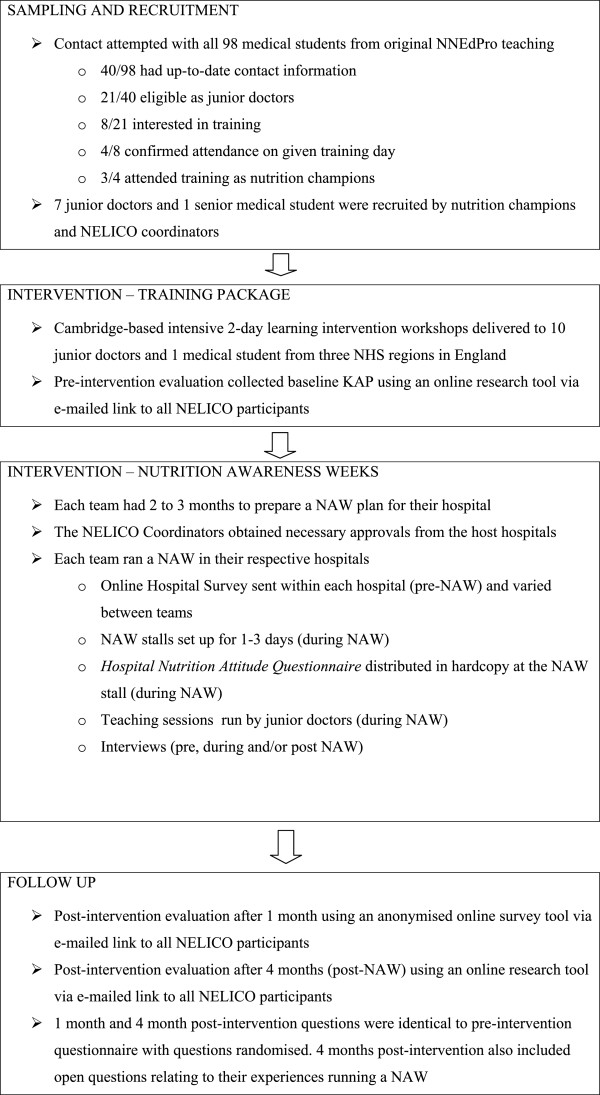
Flow chart of junior doctor recruitment and training assessment.

Recruitment to NELICO began in October 2011, via e-mail, text messaging, social media and postal correspondence with the 98 medical students who attended the original NNEdPro teaching weekends in 2009 [6]. These students had provided consent for their contact details to be securely held and to be contacted in the future [6]. Each confirmed participant (henceforth termed ‘nutrition champion’) was asked to recruit two to three junior doctors (Foundation Year 1 or 2) to form one team per hospital. These teams attended a training workshop in Cambridge before returning to their respective hospitals to run a NAW. The training workshop had a capacity of 15, as a bursary was provided to cover travel and accommodation expenses. Three teams from three hospitals (henceforth termed hospitals A, B and C) attended the training. A flow chart of events is shown in Figure 1.

### Teaching

A short module on clinical and public health nutrition was provided as a refresher for the nutrition champions and for the benefit of the newly recruited participants. Prior to teaching, participants received notes from the original nutrition training as a comprehensive reference. The remainder of the training weekend focused on organisational management and leadership strategies in the NHS and included time to start planning the NAWs.

The multidisciplinary team (MDT) of tutors consisted of three doctors, two dietitians, and three specialists in leadership and change management. The programme is shown in Additional file 1. The interactive sessions provided participants with opportunities for questions and discussions. The teaching emphasised the importance of using existing hospital nutrition resources to assist each team with their NAW, including the importance of collaboration with the hospital dietetics department.

### Evaluation of junior doctors

A 20-question pre-training questionnaire of nutrition-related KAP was sent through an anonymous online survey after participants agreed to attend the training and at one and four-months post-training (Additional file 2). Weighted average score for KAP at baseline was compared with the score at four months post-training. The weighting ensured that the three domains were each considered adequately and appropriately. Paired 2-tailed t-tests (and their non-parametric equivalent where distributions were skewed) were used to determine pre/post significance in the change to KAP scores. Post training impact in this project was simply a quality assurance measure to check if there were any major gaps in how the junior doctor received key messages from their intensive training weekend. The four-month post-training questionnaire also related to their experiences during the NAW and the whole project. Qualitative feedback was obtained through interviews and open questions added to the online pre- and post-surveys.

### Preparation for the nutrition awareness weeks

During the final afternoon session, each team created an action plan for their NAW, which were presented to the teaching group. The tutors and other participants provided feedback on the feasibility of each plan. Each team was allocated two to three months to plan their NAW. Standardised support was provided centrally by the NELICO coordinators, with additional support provided as required. Preparatory activities for the NAWs are shown in Table 1.

**Table 1:** Tasks for the preparation of the Nutrition Awareness Weeks

**Tasks**	
1	Obtaining support from senior hospital staff
2	Meeting with other nutrition representatives in the hospital
3	Choosing events
4	Booking rooms
5	Advertising campaign
6	Setting up meetings between key hospital staff and NELICO Coordinators (site visits conducted during the NAWs).

The NELICO coordinators prearranged authorisation for the NAWs from the hospitals involved and planned a site visit during the NAW to meet with the junior doctor teams and key hospital staff to determine the impact of the week. To determine impact, discussions were held between NELICO coordinators and key NHS staff during these site visit (see complete list of activities in Table 2). The objective of these visits was to understand the perceptions of hospital staff regarding:

**Table 2:** Activities held in nutrition awareness weeks in Hospital A, B and C

**Nutrition Awareness Weeks Event**	**Hospital A**	**Hospital B**	**Hospital C**
**‘MUST’ Audit**	50 patients audited regarding use of ‘MUST’	Audit conducted on ‘MUST’ use in 4 wards (results not available before publication)	70 medical patients audited regarding use of ‘MUST’
**Training for Junior Doctors**	1 hour junior doctor-led training session presented to 16 junior doctors by nutrition champion, junior doctor team and dietitians, held after mandatory training session	Case study and teaching session presented by dietetics team and other involved in hospital nutrition	2 hour junior doctor-led training session presented to 15 junior doctors by nutrition champion, and dietitians
		Presented data from emailed medical staff e-survey to junior doctors*	
**Nutrition Awareness Stall**	Stall created by junior doctor team with assistance from dietetics team	Stall created by junior doctor team with assistance from dietetics team	Stall created by junior doctor team with assistance from dietetics team
	Set up beside the main canteen and manned by a rotation of junior doctors, dietitians and NELICO coordinators for 3 days	Manned by junior doctors for 2–3 hours at lunch near the canteen on 2 days	Unmanned stall for 5 days in front of hospital canteen
	Food and supplement samples available to try	Pens and key-chains were distributed	
		Supplement samples on display	
**Staff Survey (collected at NAW stall)**	Collected from 60 staff, volunteers, visitors etc., at the stall – results analysed	Survey completed by 31 staff (17 doctors, 11 nurses and 3 other) – results analysed	Staff survey completed by 4 junior doctors – results not analysed
**Doctors Survey**	Survey sent by local NELICO team and completed by 11 junior doctors	Survey sent by local NELICO team and completed by 25 doctors and 10 nurses*	Survey sent by junior doctors and completed by 46 staff (including 19 consultants)
**Meetings: Coordinators and key hospital staff**	Hospital staff involved in nutrition, dietetics, nursing and catering	Hospital staff involved in nutrition, dietetics and nursing	Hospital staff involved in nutrition, dietetics and nursing
**Advertising**	Flyers	Flyers	Flyers
	Advert on hospital TV	Pens and key chains distributed from NAW stall	Training and NAW Stall announced at Grand Round
	E-mails to all FY1 and FY2 doctors regarding the teaching and NAW stall		

*Results of Hospital B staff e-survey were shared only with staff who attended training and were not available for this report.

• The importance of nutrition.

• The impact of a nutrition awareness week (NAW).

• The strengths and limitations of running these NAWs via junior doctors who have received a small but intense amount of training in nutrition and leadership.

To add consistency, a series of questions were used as the basis for each semi-structured interview when the representative NNEdPro members varied between site visits.

### Audit (Pre-NAW)

Before the NAW, the hospital teams set up clinical audits on a specific topic, ie the use of ‘MUST’ by ward staff, in order to gain insight into the baseline levels of nutrition awareness amongst NHS staff. With permission from the local audit office, the hospital teams completed the audits. This was a useful experience for junior doctors and a requirement of their e-portfolio (a collection of evidence that demonstrates learning achievements and abilities). Preliminary audit results were presented at the teaching sessions in each hospital and in some cases, at the NAW stalls. Further information is not provided as the emphasis in this paper is the junior doctor experience rather than the audit findings. Additionally, re-auditing was not possible during the timeframe, thus making the audit beneficial in terms of junior doctor experience but did not have a large impact on the overall effectiveness the NAWs.

### Online hospital survey (Pre-NAW)

Following training, each hospital team created a unique Online Hospital Survey (OHS) tool that was sent to all junior doctors and some other health care professionals (HCPs) through a specific e-mail list which varied within each hospital. This was sent before the NAW, with the aim of collecting information which could be used to direct the content in the teaching sessions. For example, if the majority of respondents answered incorrectly to a question relating to referral to a dietitian, this became the focus of the teaching. As each hospital produced their own survey, the questions varied between hospitals thus are not comparable.

### Hospital nutrition attitudes questionnaire (During NAW)

At each NAW stall, the Hospital Nutrition Attitudes Questionnaire (HNAQ), a 10 question multiple choice questionnaire regarding attitudes towards hospital nutrition was distributed. All hospital teams distributed the same questionnaire. This survey was designed to be completed by HCPs, however as the NAW stalls were positioned in public areas, rather than staff lounges, all hospitals received responses from a variety of respondents, including doctors, nurses, patients, volunteers etc. For this reason, it was difficult to categorise the level of nutrition knowledge and this categorising greatly limited the sample size. Based on these observations, the results of this attitude questionnaire are not shown as there is limited comparability within the responses.

## Results

### Recruitment

Three nutrition champions from three hospitals recruited junior doctors to make one team of three (hospital A) which also included one senior medical student and two teams of four (hospitals B and C), totaling 11 participants. After the training, hospital B recruited two additional junior doctors to their team to assist with the NAW.

### Training of junior doctors

**Figure 2:**
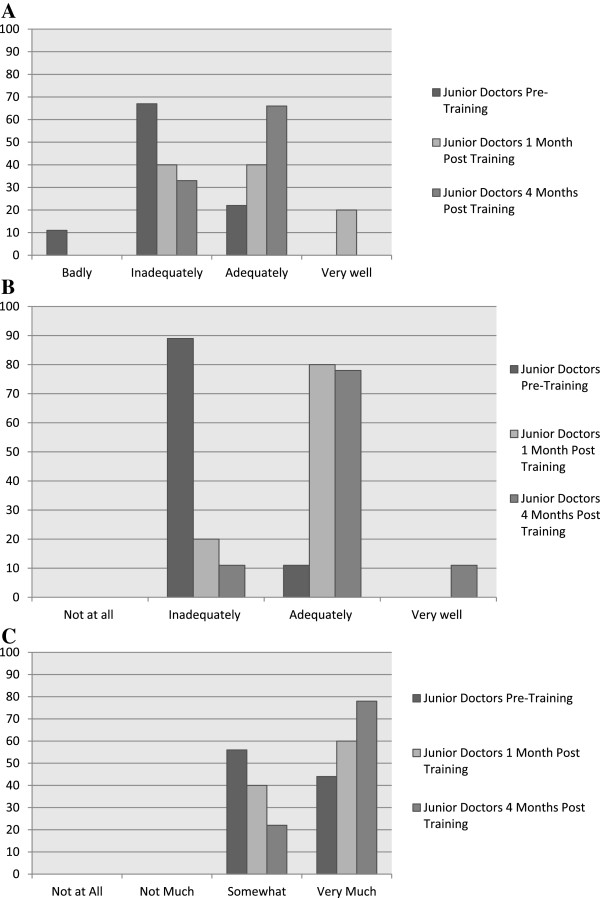
**Attitude towards hospital malnutrition as collected through questionnaire distributed at pre (n = 9) and post (1 (n = 5) and 4 months (n = 9)) training for junior doctors.** Percent responses. **(A)** How well do you think nutritional problems are managed in a hospital setting based on your experience so far? **(B)** Would you feel equipped to give general nutritional advice to patients where appropriate or required? **(C)** Do you think patients would value general nutritional advice from a doctor?

The results of a multiple choice questionnaire which had some open questions and which was completed before and one and four months after training are shown in Table 3. The high baseline scores (75%), when coupled with the success of the NAWs, suggest that a selective recruitment process is important in order to have motivated and knowledgeable junior doctors in the project. When the weighted average score for KAP at baseline was compared with the score at four months post-training, there was an increase in the overall KAP score (p = 0.03). When baseline values were compared individually for KAP with four months post-training, results were not statistically significantly different from each other. These results provide a measure of quality assurance for the initial training however the small sample size is a limitation. Additional file 2 includes the questionnaire, while Figure 2 provides more detailed information regarding three of the attitude responses provided by the junior doctors in the pre and one and four months post training questionnaire.

**Table 3:** Participants with correct scores in Knowledge, Attitudes, and Practices (KAP) for baseline, and 1 and 4 months post-training

	**Baseline (%) n = 9**	**1 Month Post-Intervention (%) n = 6**	**4 Months Post-Intervention (%) n = 9**
**Knowledge**	63	80	78
**Attitudes**	78	80	78
**Practice**	89	95	97
**Weighted Average (KAP)**	75	81	83*

*Significant between Baseline and 4 months post (p = 0.03).

Feedback using multiple choice and open-ended questions relating to the course in general (not the KAP evaluation) were collected after the training weekend and reported they all enjoyed and were engaged with the training and were keen to apply knowledge in their hospital. An example of a participant response was “*Excellent – well organised, very well-looked after by organisers. Enjoyed it very much and thought content was relevant and beneficial for junior doctors (opportunities for audits, presentations, teaching etc.)”.*

Open questions asked to participants suggest improvements for future training. Responses included, for example, making the change management section more specific to their needs: *“Very good presentations but focus on the relevant aspects of management that would be applicable to use for our upcoming venture”.*

The overall impression of the training and ratings of specific teaching sessions were recorded with questions completed on a scale that ranged from one (very poor) to five (excellent). The questions and corresponding scores are provided in Additional file 3. The overall average score for all sessions was 4.5/5. All who responded indicated they would recommend the training to a colleague.

### Nutrition awareness weeks

#### ***Hospital A***

The junior doctor team/nutrition champions successfully ran all planned events (marketing, awareness stall, teaching session etc.), coordinated effectively with the dietetics team and gained support from hospital management. Despite having only recently rotated to this hospital, the team rapidly developed key contacts and hospital support from a wide variety of hospital staff. In particular, they garnered enthusiastic support from the dietetics team at an early stage.The team from hospital A had a simple and ultimately effective action plan. As a ‘marketing strategy’, hospital A placed posters around the hospital, an advert on the hospital television network and e-mails were sent to all Foundation Year 1 and 2 doctors to promote the events that would be conducted as part of the NAW. As per Figure 1, before the NAW, the team created their own OHS relating to nutrition KAP and it was completed by 11 junior doctors. This survey highlighted that 64% of junior doctors surveyed had never made a dietitian referral, and this was used as an important factor in the development of the teaching material.

During the week, the results of the OHS and explanations of the correct answers were presented during a junior doctor-led teaching session. The team, supported by the dietetics team, conducted this non-mandatory education session, which was attended by approximately 16 junior doctors. Further discussion during teaching determined that the reason they had not made a referral was that many did not know how and when to make this referral. From the dietitians’ perspective, the week was particularly successful because junior doctors became aware of how and when to refer their patients. Although there was no pre-and post-evaluation of this session, one participant indicated that it was the *“type of practical information they need for the ward”.*

During the week, a NAW stall was created with assistance from the dietetics teams and manned by junior doctors, dietitians and NELICO coordinators for three days. The stall attracted a large number of people (n = 60, including doctors, medical student, patients, volunteers etc.) many of whom completed the HNAS which was the same as in the other hospitals involved.

#### ***Hospital B***

Hospital B was ambitious in its action plan, with several junior doctor-led teaching and awareness raising events, an advertising campaign (T-shirts, radio adverts and pens) and three NAW stalls. The teams were successful in running some, but not all of the planned activities. Thirty-one staff, including 17 doctors and 11 nurses, completed their version of the OHS. This team used the results to attract junior doctors and other interested parties to their awareness raising event. For one of their events they recruited additional hospital staff, including dietitians to assist in the delivery of the teaching, which was attended by 20 – 25 people (junior doctors, nurses and other hospital staff). At this event, they covered the questions raised by their OHS. There was also a presentation by a Nutrition Nurse Specialist who presented a case study which one NELICO junior doctor described as: *‘The talk went so well that no-one wanted to leave. All were very enthralled that the case study was from this hospital’.*

The advertising campaign was less substantial than planned and the radio advertising campaign, and T-shirts proved logistically challenging. However, the team raised funds within their group to design and order NAW pens and key-chains which were distributed throughout their NAW. At the NAW stall, 31 people (including doctors, patients etc.) completed the HNAS. Some issues in communication arose within this team during their preparations and some decisions were made without consultation of the entire team. This led to some members feeling under-valued which highlighted the importance of communication and working as part of a team when organising a NAW. Overall, interviews with the team and key hospital staff revealed that each felt an effective NAW was conducted in hospital B.

#### ***Hospital C***

Hospital C placed their focus on increasing knowledge of ‘MUST’ scores amongst junior doctors. Before the NAW, posters were created and arrangements were made to present at Grand Round during their NAW. They also created and sent their OHS, which had a high response rate, being completed by 46 doctors, including 19 consultants. As hospital C received the most comprehensive response to this survey, the main points presented in Table 4. During the NAW, results of their OHS were presented at the main event which was a voluntary nutrition teaching session for 15 junior doctors. This was run in collaboration with the dietetics team and received positive feedback through discussions with students and tutors.

**Table 4:** Main points from survey of 46 doctors at Hospital C

1	Nearly all (96%) doctors know a ‘MUST’ assessment must always be done on admission and weekly thereafter (78%), even in obese patients (100%).
2	51% of the doctors know the true purpose of the ‘MUST’ screening and 52% how prevalent malnutrition is on admission in the UK.
3	About 34% of the doctors can calculate a ‘MUST’ score themselves to verify its accuracy and 20% know where to find it in the notes in their own hospital.

For the week, the team also promoted the importance of nutrition and their teaching event at a hospital ‘Grand Round’ for doctors. The team also created a NAW stall, however due to the emphasis placed on the online questionnaire, the HNAS was not promoted, thus only completed by four people. Good networking skills were apparent in this team, as was their ability to work across a hospital with two sites.

### Impact of nutrition awareness weeks

The impact of these weeks was determined based on attendance at nutrition events, response from the OHS (hospital specific and used for the teaching) and the HNAS (created by NNEdPro and used at NAW stalls) and through semi-structured interviews with participants and key hospital staff (listed in Table 2). Attendance at all non-mandatory teaching events was better than expected by the trainers, and interview feedback showed a positive opinion from those involved.

Participants and hospital staff alike concluded it was effective to have junior doctors teaching their peers, as long as they were supported by the dietetics team. The participants attending the teaching felt it was valuable and they were more open to asking questions, particularly because they felt comfortable enough to ask the questions which they felt they should already know the answer to. At the teaching session, dietitians were particularly beneficial to provide support and answer specific nutrition-related questions.

Team members, particularly the nutrition champions, felt that they obtained beneficial skills which positively impacted on their professional development. The NELICO project provided junior doctors with the opportunity to improve their oral and verbal communication by contacting relevant hospital staff and teaching their peers. They improved their teamwork, leadership and management skills based on the theories provided in the teaching, and through working as part of a MDT. They also gained experience conducting an audit. These generic skills are applicable for the e-portfolio which is mandatory as a demonstration of continuing professional development/competence to practice. A NELICO nutrition champion was awarded joint second place at the regional annual award ceremony for junior doctor teaching, thus highlighting the significance of this work for career development.

## Discussion

The NELICO project appears to have played an important role in the professional development of a small group of tomorrow’s doctors and may be a contributing factor in the improvement of patient care by raising awareness of nutrition in hospital. The NELICO project is a novel approach to address the underlying education issues relating to hospital malnutrition, particularly due to the inclusion of change management training alongside nutrition education for the junior doctor educators. In 2013, the Francis report provided recommendations for improving care in hospitals. Education was listed as important, particularly in the recommendation which indicated the need to *‘Enhance the recruitment, education, training and support of all the key contributors to the provision of healthcare, but in particular those in nursing and leadership positions, to integrate the essential shared values of the common culture into everything they do’.*[10] The NELICO project also plays an important role to fill the gap indicated by Leach et al. in 2013, stating that NICE guidelines for nutritional screening, dietary requirements and care of people identified as at risk, are often not met [5]. Placing emphasis on tomorrow’s doctors, can help to work towards a stronger, and more nutritionally aware, patient focused, healthcare workforce.

### Junior doctors as nutrition champions

A key question discussed during the NAW site visits was whether using junior doctors as trainers and nutrition champions was an effective approach. The majority of opinions reflected that it was beneficial to emphasise these skills early in a doctors’ career. A Head of Nutrition and Dietetics Service indicated: “*Yes [it is a good approach], it seemed to help us [the dietetics team] by adding to awareness levels among doctors and interest [levels]”.* However, the opinion of one Nutrition Nurse Specialist was that *“junior doctors don’t have enough voice to be champions”*. This opinion was rare within the feedback, but raised the point that this project may detract from their mandatory work. When questioned on this point, junior doctors emphasised the added benefits to their career and personal development through involvement in NELICO. The junior doctor teams were very enthusiastic about this project and were willing to put as much time and effort into this project as possible, in accordance with their clinical work commitments. Although time constraints were mentioned as a key concern, they were outweighed by the benefits of the project.

### Hospital support

The NELICO coordinators anticipated that some hospital staff may resist a junior doctor-led project. However, there was good management support from hospitals and junior doctors were shown to have enough ‘clout’ to conduct the NAWs. Hospital A placed more focus on joining with the dietetics team to provide support for specific aspects of the week. Hospital B identified the need to recruit additional junior doctors and placed less focus on hospital management support, although further support was still obtained via the NELICO coordinators. Hospital C obtained support from key hospital management personnel, but found that working specifically with nutrition action teams provided more applied support.

The approach of using top-down buy-in from NELICO coordinators combined with the bottom-up approach from junior doctors was expected to be a barrier. However, it was found that the teams, each using a different approach, were all able to receive support from their hospital. It is unclear if using less strict recruitment criteria would produce the same result. The junior doctor participants also indicated many benefits to learning about the clinical leadership, team management and change management.

Based on the NAW interviews, all hospitals were interested in improving nutrition and were willing to put varying degrees of support into nutrition-related initiatives. This is reassuring, and may be partially as a response to the 2010 Care Quality Commission (CQC) requirements, specifically *Outcome 5: Meeting Nutritional Needs*. This requirement has brought nutrition higher up the agenda within the hospital, and this was reflected in the responses provided in the NAW interviews.

### Leadership, management and multi-disciplinary teams

The development and application of leadership and management skills are important for a junior doctor in all areas of their work. Working with managers in the hospital is one component of working within a MDT. Although the junior doctors were leaders to run the NAW, it was important for them to use other resources available within the hospital, as was highlighted during their training. Emphasis was placed on working with the dietetics team, although support was also required from other staff, particularly hospital management. Working as part of a MDT was included in the training because it is mentioned in most literature, including the 2012 BAPEN recommendation based on the NICE Nutrition Support Guidelines and best practice, indicating that *‘MDTs are needed to ensure that care pathways are appropriate and followed’*[11].

Another initiative in England, ‘Pairing trainee managers and doctors – an initiative to facilitate joint working for better patient care in the South East Coast’ [12], paired management trainees in their first year of training in the NHS with Foundation Year 2 doctors. The pair undertook a joint project to improve patient care. This project emphasised the importance of developing leadership and a greater understanding of other healthcare professions, early in their career [12].

### Economic outcomes

Due to the high annual public expenditure on disease-related malnutrition in the UK (>£13billion) [4,13], it would have been extremely useful, as a secondary aim to determine if the NELICO projects had any impact on this cost. However, the difficulty in measurement, and the lack of an accepted quantitative statistic made it unrealistic to quantitatively assess the impact of the NAWs with regard to potential cost-savings in hospital. However, the NELICO project did gain valuable qualitative evidence which can be used to shape an outcomes-based and sustainable programme that may have a direct impact on patient outcomes.

Due to the ‘generalist’ nature of ‘MUST’, it is a good indicator of engagement with the nutritional care process across different HCPs (i.e. doctors, nurses, dietitians, and other ward staff) who need to work together as a MDT to deliver nutritional care. Conducting a ‘MUST’ audit, allowed junior doctors to familiarise themselves with the tool and to raise awareness of it by sharing their results with the other staff. Increasing awareness of the ‘MUST’ score and the appropriate care plans within the hospital may ultimately benefit patient care and safety.

### Translation to policy

**Figure 3:**
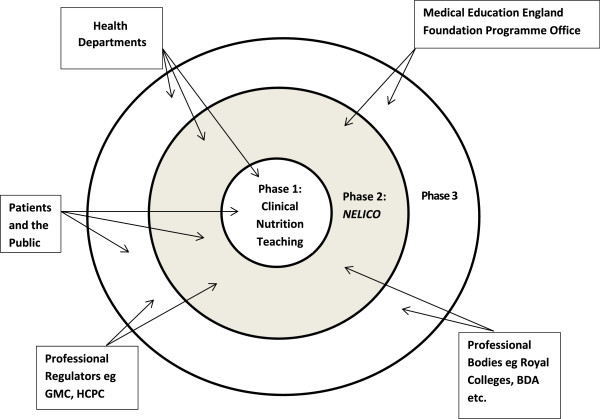
Translational model for sustained impact.

It is important that we translate the NNEdPro Group’s efforts of improving the management of clinical nutrition as a means to improve clinical outcomes and impact national policy [14]. Figure 3 outlines a translational model for sustained impact, incorporating three phases of NNEdPro and examples of key organisations that are stakeholders in this process, including the Department of Health, Health Education England, Foundation Programme Office, professional regulators (e.g. General Medical Council (GMC), Health and Care Professions Council), professional bodies (e.g. Royal Colleges), and most importantly, the patients. Understanding change management techniques within the NHS is particularly important following all of the changes to NHS structure which took effect in spring 2013. Tomorrow’s doctors will need to accommodate for the changing work practices.

Translation to policy can also be achieved on a smaller scale, at the hospital and deanery level. Based on the success of this project, we are exploring the possibility of this training integrated into mandatory NHS training potentially via the Foundation Programme, and exploring the option of offering this training across the UK.

The NELICO project has highlighted that the ‘need for nutrition education’ is associated with the ‘need for nutrition regulation’. Establishing a regulatory framework that mandates the safe and effective delivery of nutritional care in hospitals by doctors and other HCPs is a key policy objective that needs to be addressed. Such regulation needs to be at the institutional (e.g. CQC requirements) and at the individual level (e.g. regulators such as the GMC). NELICO has developed a dialogue on the importance of regulation in nutrition within the UK Inter-Regulatory group hosted by the GMC.

### Lessons learned

This project was our first attempt to run NAWs and train junior doctors in this method through the change management and leadership education. The group learned many lessons which can be incorporated into future work. The outcome of this project indicates that junior doctors should be part of a MDT involved in integrating the importance of nutrition into the hospital environment. They have the enthusiasm to be nutrition champions but can lack the time availability required. Less focus could be placed on having one NAW and more effort into having junior doctors working within a variety of specialities to incorporate nutrition into existing projects and events which may lead to more sustainability of the project.

If these NAWs were to be run again, it may be advantageous to allow for a more strategic positioning of the weeks (i.e. based on availability of junior doctors, adding nutrition into other awareness weeks) which may lead to a greater impact. Stronger consideration should also be given to the time constraints of junior doctors. A limitation of this work was the limited comparability between hospitals, particularly as each hospital created their own OHS and the HNAS was not always applicable to the audience to which it was delivered. Audit and surveys should have also been standardised across hospitals to ensure comparability and re-audits should be more encouraged, along with longer term follow-up of the event. It would have been beneficial to include pre- and post-training evaluations on KAP for the junior doctor-led training to quantify effectiveness of the teaching. Several suggestions from these weeks can also be incorporated, for example, one junior doctor suggested participants could create key learning points from their weeks which would be given to future participants. In future work, increased focus could also be placed on the impact of patient care and safety, and/or determining the cost effectiveness of improving nutrition awareness. The impact of the management and leadership teaching component should also be more thoroughly evaluated.

## Conclusion

Based on the lessons learned to date from the NELICO project, it is evident that junior doctors can have the enthusiasm, and drive to be champions in promoting nutrition awareness. This dispels the notion that bringing about changes to nutritional care needs to be a top-down process. We feel that it is essential to build on the success and innovation of the NELICO project through testing scalability, effectiveness and continuing to work towards expanding and integrating its principles into mandatory NHS training. Whilst we will continue to focus on increasing nutrition awareness in hospitals to improve patient care and safety, we believe that knowledge is essential, but the addition of leadership, change management and communication skills are key to improving the sustainable delivery of optimal nutritional care. This will work towards making a stronger, patient focused and a more cost-effective NHS.

## Abbreviations

NNEdPro: Need for Nutrition Education/Innovation Programme; NAW: Nutrition awareness week; NELICO: Nutrition Education for Improving Clinical Outcomes; NHS: National health service; BAPEN: British Association for Parenteral and Enteral Nutrition; MUST: Malnutrition Universal Screening Tool; KAP: Knowledge, Attitude and Practice; MDT: Multi-disciplinary teams; CQC: Care Quality Commission; HCPC: Health and Care Professions Council.

## Competing interests

All authors have completed the ICMJE uniform disclosure form at http://www.icmje.org/coi_disclosure.pdf and declare: no support from any organisation for the submitted work; no financial relationships with any organisations that might have an interest in the submitted work in the previous three years; no other relationships or activities that could appear to have influenced the submitted work.

## Authors’ contributions

All authors are NNEdPro Group members and contributed to all aspects of this manuscript. SR is founder/chairman; CL is managing coordinator/analyst; PD is vice chairman; MRR is medical advisor; MVE was programme manager; SG is an advisor; and JG is lead scientific advisor. SR, CL, PD, MRR, MVE, SG and JG were involved in all aspects of the NELICO project including initial preparation, recruitment, course teaching, and awareness weeks. JR, TE and MS were involved with teaching, data collection and paper writing. LM, KG, CM, and AA were junior doctor group leaders/members who ran the awareness weeks. All authors read and approved the final manuscript.

## Authors’ information

Dr Sumantra Ray and Celia Laur are the joint first author.

## Pre-publication history

The pre-publication history for this paper can be accessed here:

http://www.biomedcentral.com/1472-6920/14/109/prepub

## Supplementary Material

Additional file 1 Programme for NELICO training held in Cambridge in March 2012. Click here for file

Additional file 2: Pre-teaching questionnaire distributed during the junior doctor teaching. Click here for file

Additional file 3: **Participant feedback, average, maximum and minimum scores of programme quality from junior doctor participants completed at conclusion of teaching.** 1= very poor, 5 = excellent, (n=11). Click here for file
