# Supplementary material for: Nutrition education and leadership for improved clinical outcomes: training and supporting junior doctors to run ‘Nutrition Awareness Weeks’ in three NHS hospitals across England
Source: BMC Med Educ. 2014 May 29;14:109. doi: 10.1186/1472-6920-14-109 (PMC4059452; doi:10.1186/1472-6920-14-109)
Supplement: Additional file 1 — Programme for NELICO training held in Cambridge in March 2012. [file 1472-6920-14-109-S1.doc]

**Additional file 1: Programme for NELICO Training held in Cambridge in March 2012**

Day 1: Saturday March 24, 2012

| **Time** | **Activity** | **Faculty** |
| --- | --- | --- |
| 09:00 | Registration | NELICO Team* |
| 09:30 | Welcome | Sumantra Ray |
| 10:00 | Nutrition Screening vs Assessment | Jean Redmond and Tim Eden |
| 10:30 | Case Study / MUST practical | NELICO Team* |
| ***11:00*** | ***Coffee*** |  |
| 11:15 | Fluids, Electrolytes and Micronutrients | Minha Rajput-Ray |
| 12:15 | Over and Under Nutrition | Sumantra Ray |
| ***12:45*** | ***Lunch*** |  |
| 13:30 | Protected Mealtimes Debate | Jean Redmond and Tim Eden |
| 14:30 | Leadership training for non-management professionals – the basis for innovation’ | Peter Hiscocks |
| 15:00 | Leadership and Management in the NHS | Steve Gillam |
| ***16:00*** | ***Coffee*** |  |
| 16:15 | Leadership and Management in the NHS | Steve Gillam |
| ***17:30*** | ***Close of day 1*** |  |

**Dinner at St John’s College at 19:00**

* Sumantra Ray, Minha Rajput-Ray, Jean Redmond, Tim Eden, Celia Laur, Marietta Sayegh, Mary Ghasemi, and Mike van der Es

Day 2: Sunday March 25, 2012

| **Time** | **Activity** | **Faculty** |
| --- | --- | --- |
| 09:30 | Change Management | Mike van der Es |
| ***10.30*** | ***Coffee*** |  |
| 10.45 | Change Management | Mike van der Es |
| 11.30 | Workshop and Discussion |  |
| ***13:00*** | ***Lunch*** |  |
| 13:45 | Debrief | Sumantra Ray |
| 14.00 | Workshop and Discussion | Sumantra Ray |
| 14:40 | Presentations x 2 | Sumantra Ray |
| ***15:20*** | ***Coffee*** |  |
| 15:30 | Presentations | Sumantra Ray |
| 15.50 | Final Debrief | Sumantra Ray |
| ***16:30*** | ***Close of day 2*** |  |
