# Supplementary material for: Nutrition education and leadership for improved clinical outcomes: training and supporting junior doctors to run ‘Nutrition Awareness Weeks’ in three NHS hospitals across England
Source: BMC Med Educ. 2014 May 29;14:109. doi: 10.1186/1472-6920-14-109 (PMC4059452; doi:10.1186/1472-6920-14-109)
Supplement: Additional file 2: — Pre-teaching questionnaire distributed during the junior doctor teaching. [file 1472-6920-14-109-S2.doc]

**Additional file 2: Pre-teaching questionnaire distributed during the junior doctor teaching.**

***Please circle or highlight the correct answers***

1. How soon after admission should you screen for malnutrition?
2. 24 hours
3. 48 hours
4. 72 hours
5. 1 week
6. You need to organise a routine Chest X-ray for a patient. Which slot would be most appropriate?
7. 6.30-7.00 am
8. 10.30-11.00 am
9. 12.30-1.00 pm
10. 4.30-5.00 pm
11. How many litres of 5% Dextrose are needed to maintain an energy intake of 2000kcal/day?
12. 2 litres
13. 5 litres
14. 10 litres
15. 15 litres
16. When considering nutritional support for an obese patient (BMI 50 kg/m^2^) what would be the energy requirement?
    1. As per patient’s weight
    2. 500 kcal less
    3. 1000 extra kcal
    4. 1000 less kcal
17. Which bloods would you request for a stroke patient who has just been started on PEG feeding after 10 days of being nil by mouth?
    1. Magnesium, Phosphate, Potassium
    2. Liver function test
    3. Urea, Creatinine
    4. Glucose
18. A 35 year old gentleman with history of alcohol excess is admitted in confused state. Which nutritional supplement must be given?
    1. Thiamine
    2. Amino acid mix
    3. Oral nutrition supplements
    4. Multivitamin
19. A patient on your ward has a MUST score of 2 and the dietitian cannot see him until tomorrow. What can you do in the interim?
    1. Initiate nasogastric tube feeding
    2. Prescribe oral nutrition supplements
    3. Wait until tomorrow
    4. Start IV 5% dextrose
20. What are the odds that patient you are clerking is malnourished?
21. 1 in 2
22. 1 in 3
23. 1 in 5
24. 1 in 7
25. How well do you think nutritional problems are managed in a hospital setting based on your experience so far?
26. Badly
27. Inadequately
28. Adequately
29. Very well
30. As one of tomorrow’s doctors, would you feel equipped to give general nutritional advice to patients where appropriate or required?
31. Not at all equipped
32. Inadequately equipped
33. Adequately equipped
34. Very well equipped
35. Do you think patients would value general nutritional advice from a Doctor?
36. Not at all
37. Not much
38. Somewhat
39. Very much
40. Do you think that from a public health perspective, nutrition is important in reducing the global burden of disease?
41. Not at all
42. Not much
43. Somewhat
44. Very much
45. What is the best-known management tool to help you assess the external environment, and the way it will be putting pressure on your hospital, or health service.

a. Pesteli analysis c. Force field analysis

b. 7 S analysis d. SWOT analysis

1. If you wanted to map how patients with a certain type of condition get referred into the relevant outpatient clinic and how long it might take, what is the obvious tool to use?

a. 7 S analysis c. Process mapping

b. Force field analysis d. Unfreeze change Refreeze

1. What is the best way to respond to resistance you notice in staff who you want to cooperate with a change process you are spearheading?
2. Listen to their reactions, respect and support them, c. Warn them that resistance will noted

and hope to slowly bring them around. and will restrict their career.

1. Remind everyone else these staff resisting are d. Ignore them.

letting the team down.

1. Can you define management and name some theories of leadership?
2. Can you define clinical governance?
3. Please briefly describe the audit cycle.
4. How would you define ‘quality’ in health care?
5. What is the Care Quality Commission?

*Many thanks for completing this questionnaire. Are you happy for the NNEdPro team to contact you in future?*

*Yes  No *
