# Supplementary material for: Nutrition education and leadership for improved clinical outcomes: training and supporting junior doctors to run ‘Nutrition Awareness Weeks’ in three NHS hospitals across England
Source: BMC Med Educ. 2014 May 29;14:109. doi: 10.1186/1472-6920-14-109 (PMC4059452; doi:10.1186/1472-6920-14-109)
Supplement: Additional file 3: — Participant feedback, average, maximum and minimum scores of programme quality from junior doctor participants completed at conclusion of teaching. 1= very poor, 5 = excellent, (n=11). [file 1472-6920-14-109-S3.doc]

**Additional file 3:** Participant Feedback, average, maximum and minimum scores of programme quality from Junior Doctor participants completed at conclusion of teaching. 1= very poor, 5 = excellent, (n=11)

| **Feedback Questions**  **(How would you rate the…)** | **Average Score** | **Maximum Score** | **Minimum**  **Score** |
| --- | --- | --- | --- |
| Overall impression of the training | 4.8 | 5 | 4 |
| Overall content and delivery of the training | 4.5 | 5 | 4 |
| Suitability of the venue | 4.9 | 5 | 4 |
| Catering | 4.7 | 5 | 3 |
| Nutrition Screening vs Assessment session | 4.6 | 5 | 4 |
| Case Study / MUST practical session [missing = 1] | 4.7 | 5 | 4 |
| Fluids, Electrolytes and Micronutrients session | 4.0 | 5 | 4 |
| Over and Under Nutrition session | 4.5 | 5 | 4 |
| Protected Mealtimes Debate | 4.5 | 5 | 2 |
| Leadership Training for Non-management Professionals session | 4.6 | 5 | 4 |
| Two sessions on Leadership and Management in the NHS | 4.1 | 5 | 3 |
| Change Management session | 4.2 | 5 | 3 |
| Would you recommend this training to a colleague? | yes = 9, no = 0, missing = 2 | | |
